# A Calcium-Related Immune Signature in Prognosis Prediction of Patients With Glioma

**DOI:** 10.3389/fcell.2021.723103

**Published:** 2021-09-28

**Authors:** Cha Lin, Jian Chen, Zhaoying Su, Pei Liu, Zheyu Liu, Chenchen Zhu, Dan Xu, Zhongda Lin, Pei Xu, Ganqiang Liu, Xinjian Liu

**Affiliations:** ^1^Department of Biochemistry, Molecular Cancer Research Center, School of Medicine, Sun Yat-sen University, Shenzhen, China; ^2^Neurobiology Research Center, School of Medicine, Sun Yat-sen University, Shenzhen, China

**Keywords:** gliomas, calcium signal, risk score, immune signature, Prognosis prediction model

## Abstract

**Background:** Immune checkpoint inhibitors have been successfully used in a variety of tumors, however, the efficacy of immune checkpoint blockade therapy for patients with glioma is limited. In this study, we tried to clarify gene expression signatures related to the prognosis of gliomas and construct a signature to predict the survival of patients with gliomas.

**Methods:** Calcium-related differential expressed genes (DEGs) between gliomas and normal brain tissues were comprehensively analyzed in two independent databases. Univariate, multivariate Cox regression analysis and proportional hazards model were used to identify the prognostic of calcium-related risk score signature. The CIBERSORT algorithm and association analysis were carried out to evaluate the relationship between calcium-related signature and characteristic clinical features, tumor-infiltrating immune cell signatures as well as immune checkpoint molecules in glioma. A nomogram model was developed for predicting the overall survival for patients with gliomas.

**Results:** We found the intersection of 415 DEGs between gliomas and normal brain tissues, and identified that an eighteen calcium-related gene panel was significantly enriched in these DEGs. A calcium-related signature derived risk score was developed to divide patients into high- and low-risk groups. Low levels of calcium-related gene expression in high-risk score cases were accompanied with worse outcomes of patients. Calcium-related risk scores were significantly associated with characteristic clinical features, immune infiltrating signatures of tumor microenvironment, and exhausted T cell markers including programmed cell death 1 (PD-1), lymphocyte activating 3 (LAG3), and T cell membrane protein 3 (TIM-3), which contribute to an adverse therapeutic effect of immunotherapy. Calcium-related signature risk score was considered as an independent prognostic parameter to predict the of overall survival of patients with gliomas in nomogram model.

**Conclusion:** Our study demonstrated that calcium signaling pathway is highly associated with immunosuppression of gliomas and overall survival of patients. Targeting the calcium signaling pathway might be a new strategy to reverse the immunosuppressive microenvironment of gliomas and improve the efficacy of glioma immunotherapy.

## Introduction

Gliomas remain the most prevalent and incurable primary intracranial tumors of brain cancer (Chen et al., 2017; Laug et al., 2018), with an incidence of 3.2 per 100,000 population and median overall survival (OS) of approximately 16–20 months (Gilbert et al., 2014). Maximal surgical resection followed by post-surgical treatment including radiotherapy, or chemotherapy is the current standard-of-care therapy, which is far from sufficient in combating cancer development. Recent advances in cancer genetic have established that mutations in isocitrate dehydrogenase (IDH) are common in human malignancies. In glioma, IDH mutations occur in 70–80% of low-grade gliomas (LGG, grade II and III astrocytomas, oligodendrogliomas, and oligoastrocytomas), and most secondary glioblastoma (GBM), accounting for approximately 10% of all GBM (Gupta and Salunke, 2012). Conversely, IDH mutations are barely found in primary GBM. Although the presence of IDH mutations is a strong prognostic biomarker and predicts a favorable outcome with prolonged median survival in GBM, lower-grade glioma with IDH mutation tends to recur with undergone malignant transformation to a higher grade. Recent study showed that the majority of IDH-wildtype LGGs were undiagnosed GBMs (Reuss et al., 2015), and the outcomes of IDH-wildtype LGGs has been shown to be indistinguishable from that of IDH-wildtype GBM and worse than that of IDH-mutant GBM (Tabouret et al., 2016).

Although the central nervous system (CNS) has been recognized as an “immune privileged” site (Galea et al., 2007), accumulating evidence showed the brain’s immune system is highly activated and can interact with brain tumors (Grabowski et al., 2021). Effective T cells can efflux into the CNS through the α4β1 integrin (Calzascia et al., 2005), and cytokines such as nitric oxide and tumor necrosis factor can transiently open the blood-brain barrier (BBB) to mediate immune responses (Johanns et al., 2017). Gliomas create a profoundly immunosuppressive tumor environment (TME) both locally at the tumor and systemically in the body, creating many challenges that negatively impact patient well-being and efficacy of novel immunotherapeutic approaches. There is a multitude of mechanisms for neoplastic cells to evade detection and destruction by the immune system, which includes upregulation of immunosuppressive glioma cell surface factors and cytokines, tumor microenvironment hypoxia, tumor-induced T cell and natural killer (NK) cell dysfunction, M2 phenotypic transformation in glioma-associated macrophages/microglia, etc. (Grabowski et al., 2021). M2 macrophages have pro-tumoral activity through the secretion of immunosuppressive molecules such as interleukin 10 (IL-10), transforming growth factor β (TGF-β), vascular endothelial growth factor (VEGF), and matrix metalloproteinase (MMP)-2 and MMP-9 (Mantovani et al., 2002).

In the last decade, immunotherapy, a revolution in cancer treatments and therapies, has become one of the most promising strategies with the ability to penetrate the BBB. Unfortunately, the remarkable success of immune checkpoint blockade (ICB) in other malignancies such as advanced melanoma (Wolchok et al., 2013), non-small-cell lung cancer (Garon et al., 2015) has not been reproduced in gliomas, which was mainly imputed to the highly immunosuppressive milieu of both immune and tumor cells in the TME of the glioma (Lim et al., 2018). Large randomized trials have failed to recapitulate the efficacy of ICB in patients with gliomas (Zhao et al., 2019). A recent clinical trial of anti-PD-1 treatment in recurrent glioblastoma showed that only 8% of patients demonstrated objective responses (Filley et al., 2017). Therefore, new therapeutic targets in patients with glioma remain to be discovered.

In this study, we explored intersection differential expressed genes (DEGs) between glioma tumors and normal tissues from The Cancer Genome Atlas (TCGA) cohort (691 glioma tumors and 5 normal tissues) and a public RNA sequencing data described in Wang et al. (2021) (99 glioma tumors and 10 normal samples). Further analyses indicated that the calcium signaling pathway is significantly different between glioma and normal brain tissues. Then, we defined a calcium-related risk score that stratified gliomas into high- and low-risk groups. The relationship between calcium-related risk scores and characteristic clinical features, immune infiltrating signatures of TME, and the expression of immune checkpoint molecules were uncovered. Importantly, the calcium-related risk score as an independent prognostic parameter for glioma patients under standard care was evaluated.

## Materials and Methods

### Data Collection

The gene expression RNA-seq data and clinical information of 691 gliomas (contains 525 LGG cases and 166 GBM cases) and 5 normal brain tissues were downloaded from TCGA.^1^ The processed genomic data, proteome normalized data, and clinical characteristics of 99 GBM cases and 10 normal samples were downloaded from the Supplementary Data from Wang et al. (2021). Moreover, 693 mRNA sequencing data (contains 443 LGG cases and 249 GBM cases) were downloaded from the Chinese Glioma Genome Atlas (CGGA). Affymetrix microarray-based gene expression matrix was procured from the Gene Expression Omnibus (GEO^2^) database, including 268 GBM patients in accession GSE16011 and 100 GBM patients in accession GSE4271. Patients without clinical evidence were excluded from this study.

### Differential Gene Expression and Gene Set Enrichment

R package “limma” was utilized to identify the DEGs between gliomas and normal brain samples in two transcriptional datasets including the TCGA and the dataset from Wang et al. (2021). A threshold of *p*-value less than 0.01 and the absolute log2 fold change greater than 1 was used for defining DEGs. Then we selected the intersection of DEGs (iDEGs) in these 2 datasets and explored the biological functions of the iDEGs by the Kyoto Encyclopedia of Genes and Genomes (KEGG) analysis and the gene ontology (GO) analysis. Only pathways with a *p*-value less than 0.05 were considered to be significantly enriched pathways. Gene set enrichment analysis (GSEA) was applied for identifying statistically gene sets.

### Construction of the Prognosis Gene Signature

We selected iDEGs related to the calcium signaling pathway and performed univariate cox regression analysis to determine the gene with prognostic significance in the TCGA datasets. Then the Cox proportional hazards model was adopted for the construction of the optimal gene set with R package “glmnet.” Subsequently, the linear combination of gene expression weighted by regression coefficients (Coeffs) was developed to calculate the risk scores of each patient in the TCGA dataset with the following formula:


(1) $$\mathrm{risk}\,\text{score}=\sum_{i=1}\left({\text{Coeff}}_{i}\times {\text{Exp}}_{i}\right)$$

where *i* meant the 18 calcium-related genes and Exp meant the mRNA expression level.

Meanwhile, this formula was used to calculate the risk scores for the other 4 datasets of patients with gliomas. The R package “survival” and “survminer” was used to determine the optimal cut-off value of risk score to divide patients into high- or low-risk groups. The package “survivalROC” was used to evaluate the predictive efficiency of the gene signature by developing the time-dependent receiver operating characteristics (ROC) curve with area under the curve (AUC) value.

### The Comparison of the Pathologic Features Between High- and Low-Risk Groups of Patients With Gliomas

The pathologic feature information was obtained along with the transcriptome data in the TCGA and CGGA datasets. The grade of the gliomas in the TCGA database was not allowed to download anymore, so we chose the other two datasets from GEO to study the association between the risk score and the grade of gliomas instead. To illuminate the association between the risk score and the IDH-mutation and the grade of gliomas, we used the Mann-Whitney Wilcoxon test and considered a *p*-value < 0.01 as statistical significance.

### Calculation of the Immune Cell Infiltration

We used CIBERSORT (Newman et al., 2015)^3^ to estimate the relative abundance of 22 types of immune cells in gliomas from the TCGA database. CIBERSORT is a deconvolution algorithm, which uses a set of reference gene expression values as the minimum representation of each cell type, and estimates the cell composition of complex tissues through supporting vector regression based on the gene expression profiles of a large number of tumor samples (Newman et al., 2015). The detailed definition of the 22 immune cell types can be found in Newman’s publication (Newman et al., 2015). The input matrix was the mRNA expression matrix from the TCGA database. Then, the R package “reshape2” and “ggplot2” were utilized to visualize the landscape of the relative proportion of the 22 immune cells. To compare the fractions of the immune cells between the high- and low-risk groups of patients, we used the Mann-Whitney Wilcoxon test and considered *p*-value < 0.01 as the statistical significance.

### Identification of Immune-Related Gene Expression-Based Subtypes

We performed unsupervised clustering using the R package “ConsensusClusterPlus” for class discovery based on the comparison of gene expression of 2487 immune-related gene expression profile from the TCGA database. 80% item resampling, 50 resampling, and a maximum evaluated K of 6 were selected for clustering. The cumulative distribution function (CDF) and consensus heatmap were used to assess the optimal K.

### Construction and Identification of an Individualized Nomogram Model

The clinical characters considering age, chemotherapy, IDH-mutation, and risk score were selected to construct a visualized prognostic nomogram model. R packages “rms” and “survival” were utilized to predict the probability of 1-, 2-, and 3-year overall survival for patients with gliomas. The predictive capability of the nomogram was evaluated through measuring discrimination and calibration utilizing a bootstrap approach under 1,000 resampling. Discrimination was accessed via the Concordance index (C-index), which is used to evaluate the predictive value of a nomogram. The predictive ability of the nomogram model is more accurate when the C-index of the nomogram is closer to 1. Calibration curves were applied to evaluate the consistency between the predicted survival probability of the nomogram and the actually observed possibility.

## Results

### Calcium Signaling Pathway Plays an Important Role in Gliomas

**FIGURE 1:**
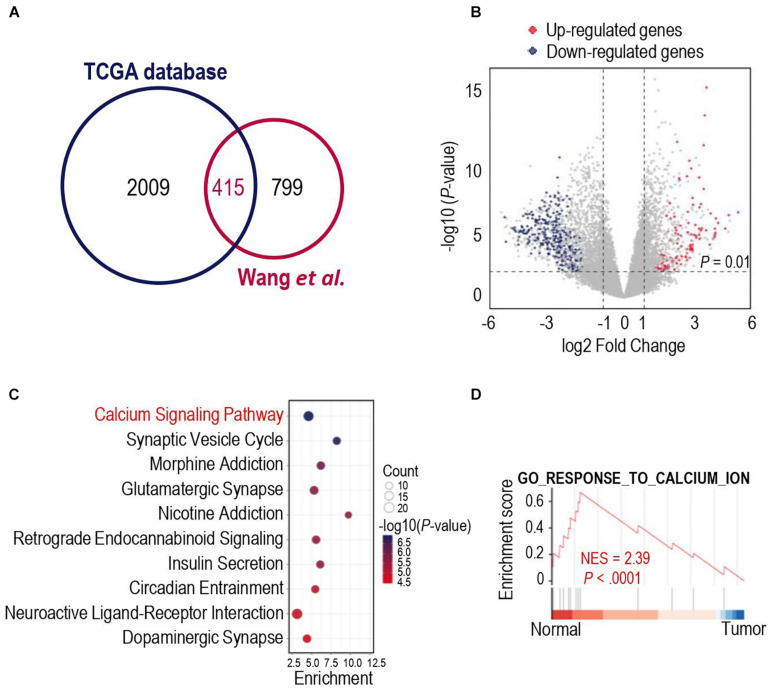
Gene expression differences in human glioma and human normal brain. **(A)** Venn diagram showed the intersected differentially expressed genes (iDEGs) from two independent datasets. **(B)** Volcano plots showed the up-regulated and down-regulated iDEGs in The Cancer Genome Atlas (TCGA) database (| log_2_Fold change| > 1 and *p-*value < 0.01). **(C)** Top 10 enriched Kyoto Encyclopedia of Genes and Genomes (KEGG) pathways by the iDEGs. **(D)** Gene set enrichment analysis (GSEA) discovered the calcium signaling pathway enriched in the normal brain cases in the TCGA database.

We first analyzed different mRNA expressions among glioma tissues and normal tissues. 2009 DEGs were detected in the TCGA database, while 799 DEGs were identified in the dataset from Wang et al. (2021). To obtained more representative DEGs, we compared these two groups of DEGs and as a result, 415 iDEGs were screened out (Figure 1A). Volcano plots showed the 112 up-regulated iDEGs and 303 down-regulated iDEGs (Figure 1B). Then KEGG and GO pathway enrichment analysis was applied for the 415 iDEGs, and found that highly expressed iDEGs in normal tissues were enriched in sets of pathways involving calcium signaling pathway (Figure 1C and Supplementary Figure 1). The calcium signal is a powerful and multifaceted tool in controlling numerous cellular processes, and the dysregulation of intracellular calcium signal can lead to tumorigenesis (Monteith et al., 2017). Gene set enrichment analysis also showed the enrichment of calcium signaling pathway in normal brain cases of the TCGA database (Figure 1D). Our results suggested that calcium signaling might contribute to the development and progression of glioma.

### Establishment of a Calcium-Related Signature and the Elevation of Its Association Between Pathologic Features

**FIGURE 2:**
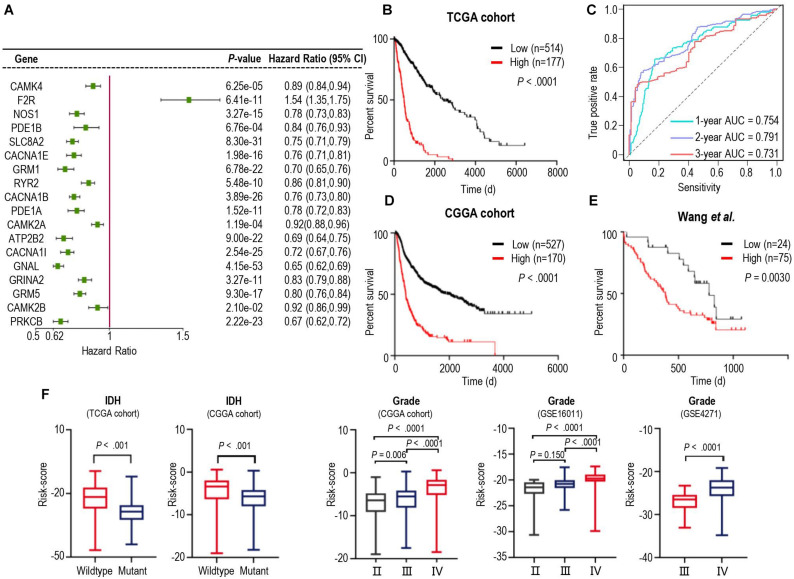
Identification of a prognostic signature by Cox proportional model in TCGA cohort and the landscape of clinical and molecular features is associated with calcium pathway-related risk-score. **(A)** Univariate Cox regression analysis showed 18 iDEGs that were significantly associated with overall survival in the TCGA dataset. **(B)** Kaplan-Meier survival curves based on the risk score in the TCGA cohort. Survival curves were compared using the log-rank Mantel-Cox test. **(C)** 1-, 2- and 3-years overall survival ROC curves in TCGA cohort demonstrated the relatively satisfactory predictive performance. **(D,E)** Survival analysis of the signature in the CGGA cohort **(D)** and dataset from Wang et al. (2021) **(E)**. **(F)** Distribution of the risk score in stratified patients from 5 different cohorts by IDH Mutation (TCGA and CGGA) and the stage of glioma (CGGA, GSE16011, and GSE4271).

We next tried to find out which iDEGs were involved in the calcium signaling pathway. As a result, 19 genes were identified (Supplementary Figure 2) and were applied Univariate Cox Regression analyses to investigate the relationship between the calcium signature and the prognosis of patients with glioma. Excluding Adenylate Cyclase 1 (ADCY1), each of the other 18 genes was significantly associated with the prognosis of the gliomas (Figure 2A). Interestingly, only Coagulation Factor II Thrombin Receptor (F2R) was significantly up-regulated in gliomas (Figure 2A), and higher F2R expression was correlated with significantly worse prognosis of cases in both TCGA and CCGA training database (Supplementary Figure 3), which is consistent with the study that demonstrated F2R was causally associated with the progression of glioma (Auvergne et al., 2016).

We then performed cross-validation tuning parameter selection by use of a proportional hazards model to confirm the association of calcium-related gene panel with the best prognostic value (Supplementary Figure 4). As a result, all eighteen calcium-related genes were identified as a classifier. The eighteen-gene panel was used to calculate the risk score of each case by computing with the gene expression level and regression coefficient. Subsequently, gliomas in training TCGA cohorts were arrayed based on risk scores and categorized into high- or low-risk groups based on an optimal cutoff value of risk scores (Supplementary Figures 5A–C). Kaplan-Meier analysis revealed that patients in the high-risk group had a significantly poorer outcome than those in the low-risk group (Figure 2B). Furthermore, we assessed the predictive accuracy by the time-dependent ROC curve and computing the AUC. The AUC values for overall survival of glioma patients were 0.754 (95% CI: 69.73–81.14) at 1 year, 0.791 (95% CI:74.58–83.64) at 2 years, and 0.731 (95% CI: 66.86–79.32) at 3 years (Figure 2C), which represented the relatively satisfactory predictive performance. We also calculated the risk scores of samples in the CGGA cohort and Wang‘s cohort with the regression Coeffs to validate this signature (Figures 2D,E). Moreover, we detected the association between this signature and IDH mutation, pathological feature of gliomas. The risk scores were distributed differently in the glioma patients stratified by IDH mutation status and grade classification (Figure 2F). In both TCGA and CGGA datasets, IDH-mutated cases generally have a lower risk score than the IDH-wildtype cases (Figure 2F). Similarly, higher levels of risk scores preferred to distribute in higher WHO grades (Figure 2F) which represented higher malignancy degree of glioma. Furthermore, the different calcium levels between high- and low-risk groups were confirmed in the proteome level by use of the proteome data from (Wang et al., 2021; Supplementary Figure 6), which were consistent with those of in the transcriptome level (Figure 2A). These results demonstrated that calcium-related signature is a strong prognostic factor in gliomas.

### Elevated Calcium-Related Risk Score Is Associated With a Higher Immunosuppressive Tumor Microenvironment in Gliomas

**FIGURE 3:**
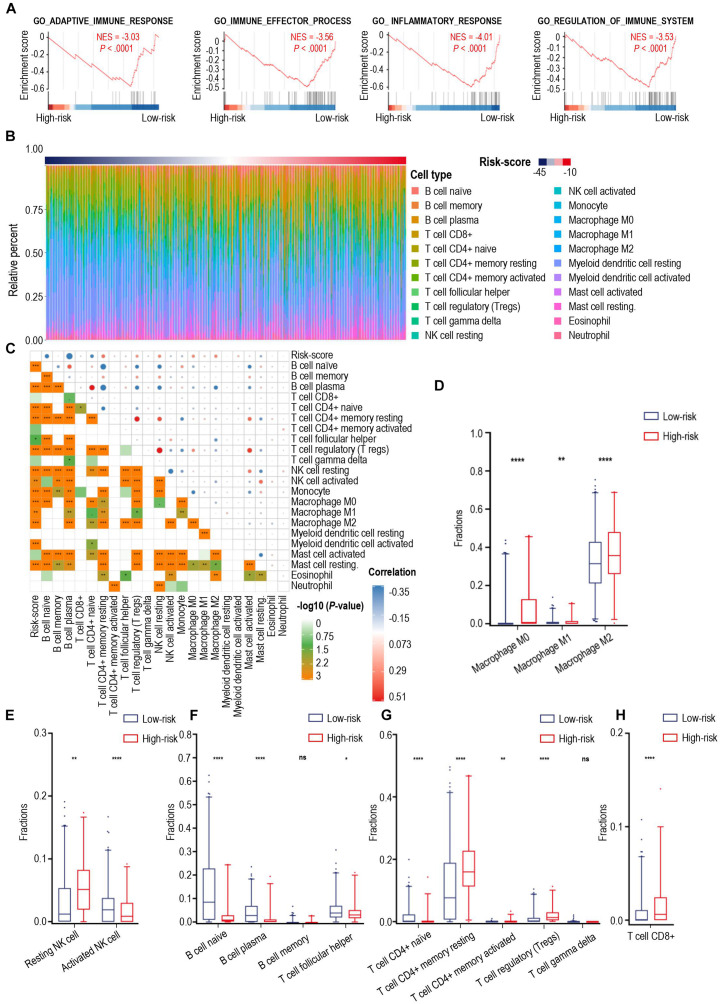
The landscape of immune infiltration in high- and low-risk patients with gliomas. **(A)** GSEA of significant immune-associated biological process between high- and low-risk patients with glioma. **(B)** The relative proportion of 22 immune cell infiltration in the TCGA cohort. Immune cell types were defined through CIBERSORT. **(C)** Pearson correlation matrix of risk score and 22 immune cell proportions including B cells, CD8^+^ T cells, CD4^+^ T cells, NK cells, monocytes, macrophages, myeloid dendritic cells, mast cells, eosinophil cells, and neutrophil cells. **(D–H)** Box-plots of fractions of macrophages **(D)**, NK cells **(E)**, B cells, T helper cells **(F)**, CD4^+^ T cells, gamma delta T cells **(G)** and CD8^+^ T cells **(H)** between high- and low-risk group. *p*-value: *, < 0.05; **, < 0.01; ****, < 0.0001; ns, not significant.

Calcium signaling serves as a ubiquitous second messenger and controls numerous cellular processes. We performed principal component analysis (PCA) for all glioma samples in the TCGA cohort and showed that the high- and low-risk score cases based on calcium-related signatures were largely consistent with discrete sections of glioma patients stratified by PCA (Supplementary Figure 5D). Then we investigated the DEGs between high- and low-risk groups and identified 2617 DEGs in the TCGA cohort and 1330 DEGs in the CGGA cohort, with 920 iDEGs of these two cohorts (Supplementary Figure 7A). Heatmap showed the expression of 682 down-regulated iDEGs and 235 down-regulated iDEGs of the high-risk group in the TCGA cohort (Supplementary Figure 7B). GO analysis showed these 920 iDEGs were highly enriched in immune response process (Supplementary Figure 8). As shown in Figure 3A, GSEA showed these 920 iDEGs were highly enriched in immune-associated pathways, including adaptive immune response (normalized enrichment score (NES = –3.03), immune effector process (NES = –3.56), inflammatory response (NES = –4.01), regulation of immune system process (NES = –3.53). Significant enrichment of immune-associated pathways in high-risk glioma cases is a paradigm shift with traditional thoughts, suggesting that the immune response of high- and low-risk groups of patients were significantly different.

We verified the association between calcium signal and immune responses in the other way. We stratified 691 gliomas patients from TCGA database and took an unbiased approach using consensus clustering of the gene expression profile. Hierarchical clustering of 2487 immune-related genes identified two main subtypes (sub1 = 440 patients, sub2 = 251 patients) (Supplementary Figure 9A), assessing by CDF curves and consensus matrices (Supplementary Figures 9B–H). Meanwhile, we found patients in sub2 had a significantly poorer clinical outcome in comparison with those in sub1 (Supplementary Figure 9I). Next, we analyzed the functional context of these two subtypes of glioma. R package “limma” was used to identify the DEGs between sub1 and sub2 and consequently, 4221 DEGs were screened out, and performed GO analysis and KEGG analysis based on the DEGs between sub1 and sub2. Our results showed that calcium signals significantly enriched in the sub1 group of gliomas (Supplementary Figures 9J,K). Heatmap showed that calcium-related genes significantly down-regulated in sub2 group of gliomas (Supplementary Figure 9L). Our results demonstrated that down-regulated-calcium signals were highly associated with the poor prognosis and immune responses of gliomas.

To explain the discrepancies of immune-associated pathways between the high-risk and low-risk groups, we investigated 22 types of tumor-infiltrating immune cells in the TCGA cohort through CIBERSORT (Figure 3B) and assessed the correlation between the immune cell infiltration and calcium-related risk scores (Figure 3C). We found risk score was significantly correlated with tumor-infiltrating immune cells including B cell naïve, B cell plasma, CD4^+^ T cells, Tregs, NK cells, macrophages (Figure 3C). Additionally, our data showed that macrophages dominantly infiltrated in all cases (Figure 3B), which are consistent to previous studies demonstrating that macrophages in tumor are a dominant immune cell type in the tumor milieu, which display an ability to suppress T cell recruitment and generally associated with a poor prognosis in solid tumors (DeNardo and Ruffell, 2019). Uncommitted macrophages (M0) are differentiated from human peripheral blood monocytes and dynamically mature under the influence of signals from the local microenvironment into either classically (M1) or alternatively (M2) activated macrophages with specific gene expression profiles, and phenotypic characteristics (Tarique et al., 2015). M1 macrophages foster inflammation response against invading pathogens and tumor cells, whereas M2 macrophages tend to exert an immune suppressive phenotype, and favoring tumor progression (Su et al., 2020). As shown in Figure 3D, the proportion of M1 macrophages are extremely low in all tested glioma cases. However, the proportion of M0, and M2 macrophages was relatively higher in the high-risk group than those in the low-risk group (Figure 3D). Low level of calcium signals-related M2 macrophages have predominantly immunosuppressive activities and was highly associated with the malignancy of glioma.

NK cells have a pivotal role in inhibiting the development of cancer due to their ability to recognize and lyse transformed cells. Elevated intracellular Ca2^+^ concentrations are required for efficient NK cell function and thus for killing their targets (Schwarz et al., 2013). Resting NK cells are generally less lytic against target cells than activated NK cells. Our data showed that the high-risk cases had significantly more resting NK cells and less activated NK cells when compared with those of low-risk cases (Figure 3E), which means the low-level expression of calcium-related genes in high-risk cases might play an important role in limiting the tumor-killing capability of NK cells.

Naïve B cells develop and differentiate to format plasma cells for secreting immunoglobulins. Moreover, B cells are critical to T-cell-mediated anti-tumoral immunity (Candolfi et al., 2011). Notably, increase in intracellular levels of calcium ions was proved to be one of the key triggering signals for B cells to secrete antigen (Baba and Kurosaki, 2016). As shown in Figure 3F, patients with low-risk scores were characterized with a significantly increased proportion of B naïve cells and plasma cells, suggesting a stronger ability to synthesize and secrete various immunoglobulins in the low-risk group. Additionally, follicular helper T cells (Tfh), which are a subset of CD4^+^ T cells and capable of inducing the differentiation of B cells into plasma cells and memory cells (Ueno et al., 2015), were found to have a higher level of infiltration in gliomas from the low-risk group of patients (Figure 3F). Furthermore, a larger fraction of CD4^+^ resting T cells and Tregs were found in high-risk cases (Figure 3G), whereas the fractions of CD4^+^ naïve T cell, CD4^+^ activated T cell, and gamma delta T cells were extremely low in the tested glioma cases. Interestingly, there were higher levels of CD8^+^ T cells in high-risk score cases in comparison with those in low-risk score cases (Figure 3H). CD8^+^ T cells are usually considered cytotoxic T cells, which are able to directly kill virus-infected cells as well as cancer cells. To explain this controversial fact, we next tried to analyze the expression of immune checkpoints in glioma.

### The Calcium-Related Risk Score Is Associated With the Expression of Immune Checkpoints

**FIGURE 4:**
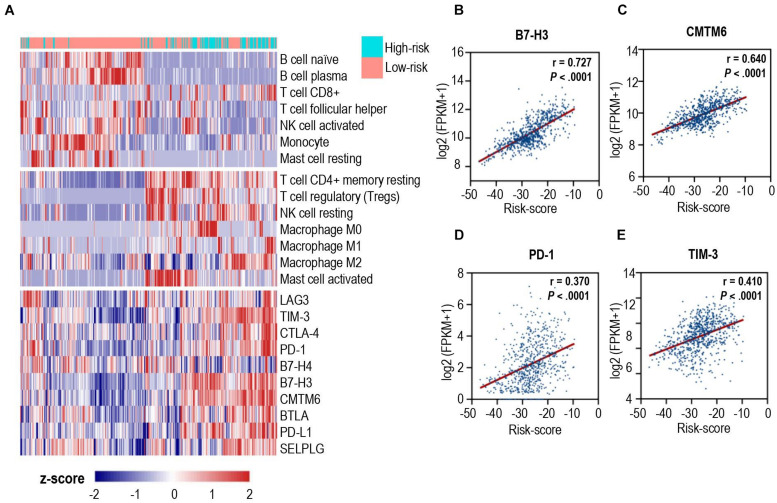
Correlation analysis between calcium-related risk score and pivotal immune checkpoints. **(A)** Heatmap showed the unsupervised cluster analysis of each immune cell proportion and immune checkpoints and calcium-related risk score in TCGA glioma cohort (*n* = 691 patients). **(B–E)** The correlation between calcium-related risk score and the expression of B7-H3 **(B)**, CMTM6 **(C)**, PD-1 **(D)** and TIM-3 **(E)**. Pearson correlation coefficient, also known as Pearson R statistical test, was shown at the top right corner of each figure.

To unravel the association between the calcium-related signatures and immune cell infiltrations, we performed an unsupervised cluster analysis (using Euclidean distances) to reconstruct calcium-related signatures of glioma in the TCGA cohort at the immune cell types level. As shown in Figure 4A, patients with low-risk scores were characterized with an increased proportion of anti-tumoral immune cells, and a decreased proportion of pro-tumoral immune cells except for CD8^+^ T cells. Next, we specially focused on the relationship between calcium-related risk score and the expression levels of crucial immune checkpoint genes, including lymphocyte activating 3 (LAG3), T cell membrane protein 3 (TIM-3), cytotoxic T-lymphocyte associated protein 4 (CTLA-4), PD-1, B- and T-lymphocyte-associated protein (BTLA), B7-H4, and B7-H3. There was a significantly positive correlation between risk score and the expression of these immune checkpoints (Lower panel of Figures 4A–E). Our data indicated that upregulation of these immune checkpoints in high-risk cases (Lower panel of Figure 4A) was tightly associated with the calcium-related risk scores (Figures 4B–E), which explained the fact that higher level of CD8^+^ T cells in high-risk cases was exhausted T cells and unable to elaborate anti-tumor function. These data demonstrated that calcium-related signature in glioma is highly associated with the profound immunosuppressive tumor microenvironment, which might limit the efficacy of immune checkpoint blockade therapy for glioblastoma.

### Construction and Identification of an Individualized Nomogram Model Based on Calcium-Related Signature

**FIGURE 5:**
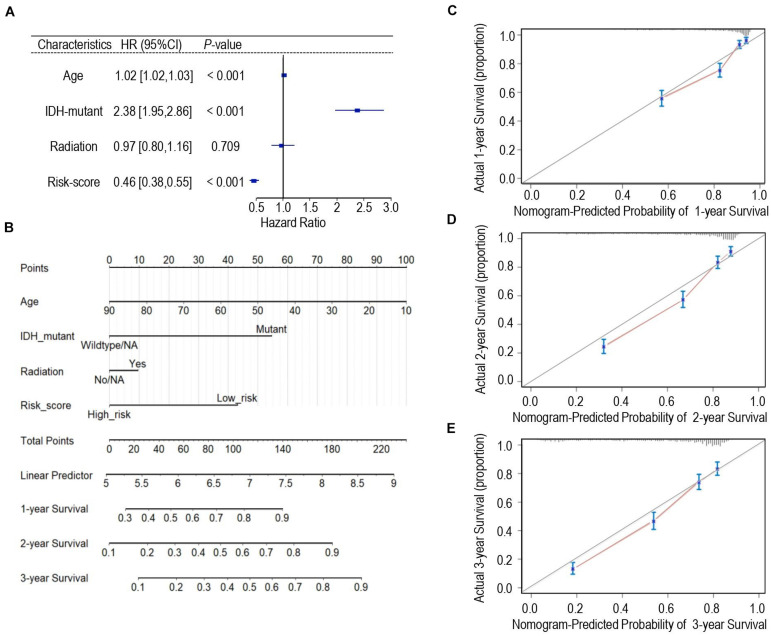
Development of the nomogram predicting the overall survival for glioma patients. **(A)** Forest plot of the multivariate Cox regression analysis indicated that the risk-score was an independent predictor for OS. **(B)** Nomogram model for predicting the probability of 1-year, 2-year, and 3-years OS in gliomas. **(C–E)** Calibration curves of the nomogram model concerning consistency between predicted and actual 1-year **(C)**, 2-year **(D)**, and 3-year **(E)** OS.

Univariate Cox regression analysis was applied for assessing the contribution of a calcium-related signature as an independent prognostic parameter to the overall survival of glioma cases in the TCGA cohort. As shown in Figure 5A, calcium-related risk score as well as other clinicopathologic parameters such as age, IDH mutation status were independent prognostic factors to affect the overall survival of glioma. Then, we carried out multivariate Cox analysis to develop a nomogram model through integrating the identified significant parameters including age, radiation, IDH-mutant, and calcium-related risk score, which might be a quantitative method to predict the survival of glioma cases. In the nomogram model, scores of each parameter were identified through plotting a straight line upward and the total points of each patient were defined by adding up the scores of each parameter (Figure 5B). For each patient with glioma, the survival rate in 1, 2, and 3 years was estimated by plotting a perpendicular line downwards from the total point axis to the resulting axis (Figure 5B). C-index, which was defined as the ratio of all patient pairs whose predictions were consistent with the results, was used to appraise discriminating ability of the nomogram. The C-index for the nomogram was 0.743 (95% CI: 0.734–0.753), and favorable calibration was also confirmed (Figures 5C–E), indicating the satisfactory consistency between nomogram prediction and practical observation in 1-, 2-, 3-year OS of glioma patients. However, the limitation of nomogram model is that we cannot take all pathologic features such as MGMT, performance status, and chemotherapy into consideration since the incomplete data provided in either TCGA or CGGA datasets.

## Discussion

In this study, we systematically evaluated the prognostic values of our calcium-related gene signature in patients with gliomas and proposed a calcium-related risk score system. The higher calcium-related risk score was tightly associated with the immunosuppressive tumor microenvironment and predicted a worse prognosis of glioma patients, suggesting that the calcium-related risk score provided an appropriate tool for pretreatment stratification and post-trial evaluation of patients.

Calcium signals represent a ubiquitous regulator that controls a variety of cellular processes (Prevarskaya et al., 2011; Marchi and Pinton, 2016), which include cell proliferation, metabolism, and gene transcription (Déliot and Constantin, 2015). The free calcium ion concentration in the cytoplasm is regulated through Ca2^+^ channels and its stores in the endoplasmic reticulum, controlling a large array of principle physiological and pathological processes (Marchi and Pinton, 2016). There are three principal entry channels for calcium influxes: the voltage-gated channels, the store-operated channels (SOC), the receptor-operated channels (ROC). Voltage-gated channels are divided into low (T-type) and high voltage (N, L, R, P/Q) channels. Previous studies have shown that T-type calcium channel blockade may inhibit cancer cell proliferation via a cytostatic mechanism (Rasmussen and Rasmussen, 1990; Munaron et al., 2004; Marchi and Pinton, 2016). It was widely proved that mibefradil, an anti-hypertensive drug that blocks T-type calcium channels (Abernethy, 1997), can inhibit the growth of human GBM xenografts, increased survival, and enhanced temozolomide sensitivity (Abernethy, 1997; Keir et al., 2013; Valerie et al., 2013; Zhang et al., 2017). Besides, blocking high voltage channels with ZnCl_2_ significantly reduced the cell survival, and migration of human GBM cell line T98G, indicating that high voltage channels can be a new therapeutic target for GBM (Ribeiro-Silva et al., 2016). In our study, we found that voltage-gated calcium genes including Calcium Voltage-Gated Channel Subunit Alpha1 B (CACNA1B) (N-type calcium channel), Calcium Voltage-Gated Channel Subunit Alpha1 E (CACNA1E) (R-type calcium channel), and Calcium Voltage-Gated Channel Subunit Alpha1 I (CACNA1I) (T-type calcium channel) are significantly down-regulated in the gliomas compare with the normal brain tissue (Supplementary Figure 2), and the down-regulated expression of these three genes are strongly associated with the poor outcomes of the patients with gliomas (Figure 2A). Besides, SOCs and ROCs are also major calcium influx routes in cells, especially for non-excitable cells. Recent research showed that major modifications appear in the regulation of the SOC mechanism in glioblastoma and glioblastoma stem cells, compared to normal brain tissues (Robil et al., 2015). Moreover, SOC inhibitors diminished proliferation and substantially reduces the self-renewal capacities of glioblastoma stem cells (Terrié et al., 2021). However, our 18 calcium-related genes did not involve in the SOCs or ROCs.

Although current studies mainly focused on the cancer cell itself as the target for modifying the calcium signals, increasing evidence showed that calcium signaling is a novel way to target the TME. In this study, we demonstrated that a calcium-related risk score was correlated with the signature of tumor-infiltrating immune cells. The elevated risk score, which represented low-level expression of calcium-related genes, was significantly associated with a higher immunosuppressive microenvironment in gliomas. In fact, the elevation of intracellular free Ca2^+^ is proved to be one of the triggering signal for T cell activation by antigen (Feske et al., 2001). CD8^+^ T cells recognize peptides presented by MHC Class I molecules, and are very important for immune defense against intracellular pathogens and for tumor surveillance. In consistent with previous studies (Chen et al., 2019), only small amount of CD8^+^ tumor-infiltrating lymphocytes exists in all tested glioma cases, although there was a slightly higher level of CD8^+^ T cells in calcium-related high-risk cases when compared those of in low-risk cases. Furthermore, we demonstrated that multiple immune checkpoints including LAG3, TIM-3, CTLA-4, PD-1, BTLA, B7-H4, and B7-H3 were highly expressed in calcium-related high-risk cases. The upregulation of these T cell exhaustion markers may synergistically inhibit CD8^+^ cytotoxic T cell function in gliomas. Our study may provide an explanation for the recent failure of the phase 3 clinical trial of anti-PD-1 treatment in recurrent glioblastoma (Filley et al., 2017; Reardon et al., 2020). Targeting the calcium signaling pathway to increase intratumoral calcium level might reverse the profound immunosuppressive microenvironment and would be beneficial for glioma immunotherapy. Taken together, our study identified a novel 18-gene signature for gliomas prognosis prediction. Our nomogram containing the calcium related signature can be used as a predictor for prognosis and has critical clinical implications, which was recognized to process more precise performance for predicting the outcomes of patients with glioma.

## Data Availability Statement

The datasets presented in this study can be found in online repositories. The names of the repository/repositories and accession number(s) can be found in the article/Supplementary Material.

## Author Contributions

XL and CL: conception and design, writing, review, and revision of the manuscript. CL, JC, ZS, PX, and GL: development of methodology. CL, PL, ZLiu, CZ, DX, and ZLin: acquisition of data. XL: study supervision. All authors contributed to the article and approved the submitted version.

## Conflict of Interest

The authors declare that the research was conducted in the absence of any commercial or financial relationships that could be construed as a potential conflict of interest.

## Publisher’s Note

All claims expressed in this article are solely those of the authors and do not necessarily represent those of their affiliated organizations, or those of the publisher, the editors and the reviewers. Any product that may be evaluated in this article, or claim that may be made by its manufacturer, is not guaranteed or endorsed by the publisher.

## Footnotes

1
https://www.genome.gov/Funded-Programs-Projects/Cancer-Genome-Atlas

2
http://www.ncbi.nlm.nih.gov/geo/

3
http://cibersort.stanford.edu/
