# Supplementary material for: A Calcium-Related Immune Signature in Prognosis Prediction of Patients With Glioma
Source: Front Cell Dev Biol. 2021 Sep 28;9:723103. doi: 10.3389/fcell.2021.723103 (PMC8505737; doi:10.3389/fcell.2021.723103)
Supplement: Supplementary file 1 [file Data_Sheet_1.PDF]

Fig. S1

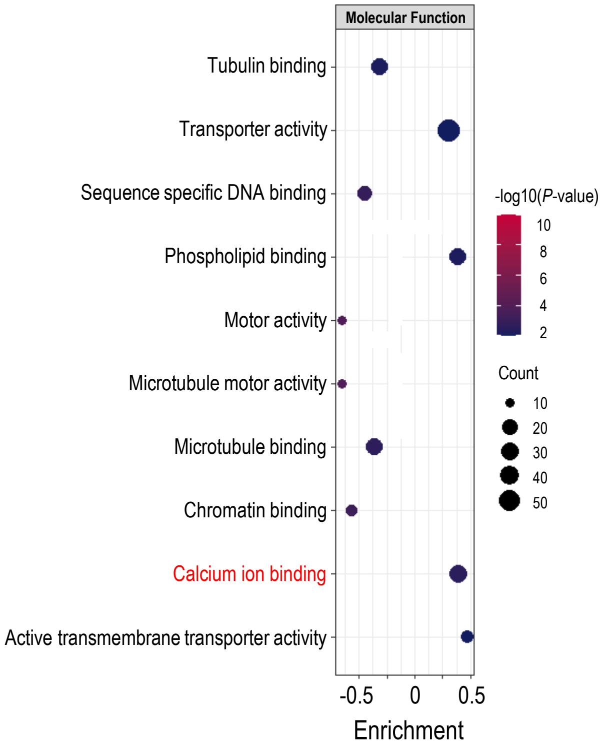

Fig. S1 GO analysis of 415 iDEGs between normal brain and glioma showed enrichment of calcium iron binding.

Fig. S2

19 calcium-related iDEGs	
CAMK4	CAMK2A
F2R	ATP2B2
NOS1	CACNA1I
PDE1B	GNAL
SLC8A2	GRIN2A
CACNA1E	GRM5
GRM1	CAMK2B
RYR2	PRKCB
CACNA1B	ADCY1
PDE1A	

Fig. S2 19 genes involving in the calcium signaling pathway from KEGG within 415 iDEGs were screened out.

Fig. S3

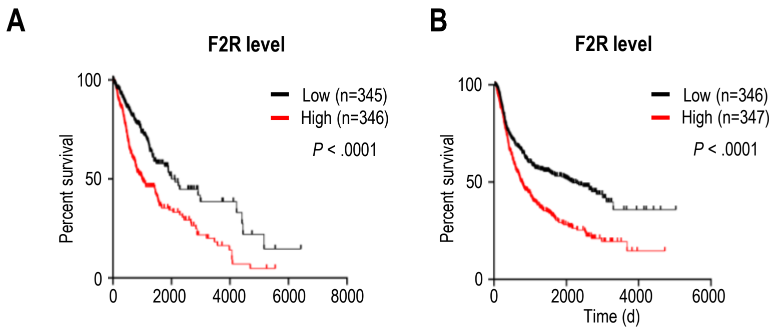

Fig. S3 Glioma patients with higher F2R levels generally had shorter overall survival in the TCGA cohort (A) and the CGGA cohort (B).

Fig. S4

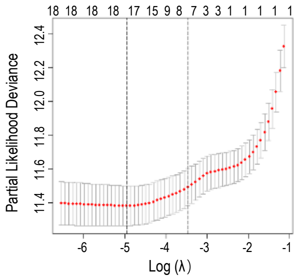

Fig. S4 Cross-validation for tuning parameter selection in the proportional hazards model.

Fig. S5

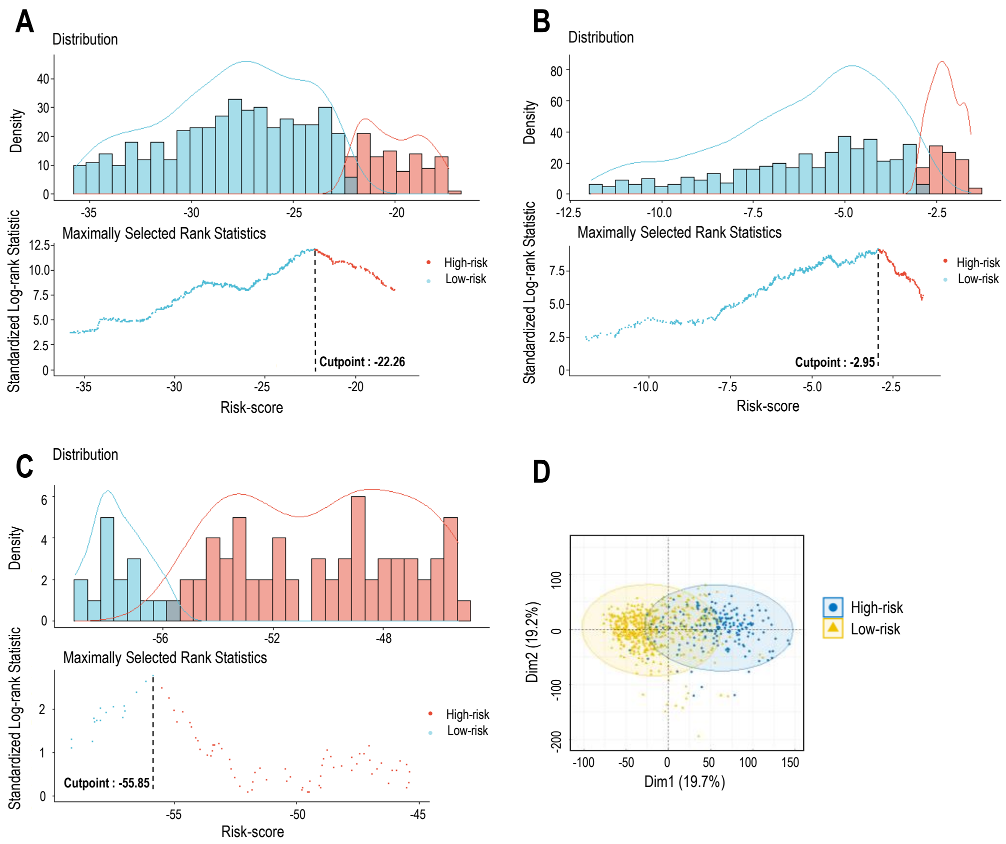

Fig. S5

(A-C) The best cut-off value of risk score in TCGA database (a), CGGA database (b), and data from Wang et al. (c) determined by the R package “survminer”. (D) Principal component analysis (PCA) plot of all glioma samples from the TCGA cohort. Samples fall into two groups separating the high-risk group and the low-risk group of samples.

Fig. S6

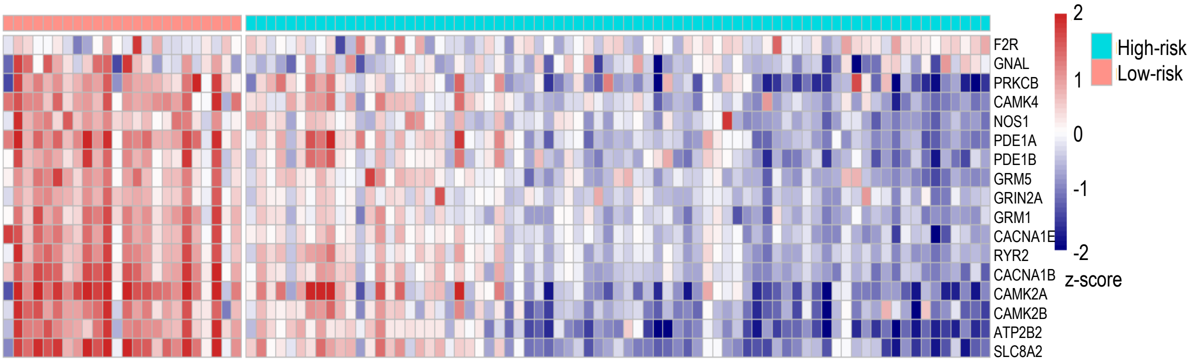

Fig. S6 Heatmap of the protein abundance by the proteome analysis showed differential expression between high- and low-risk groups in the dataset from Wang *et al*.

Fig. S7

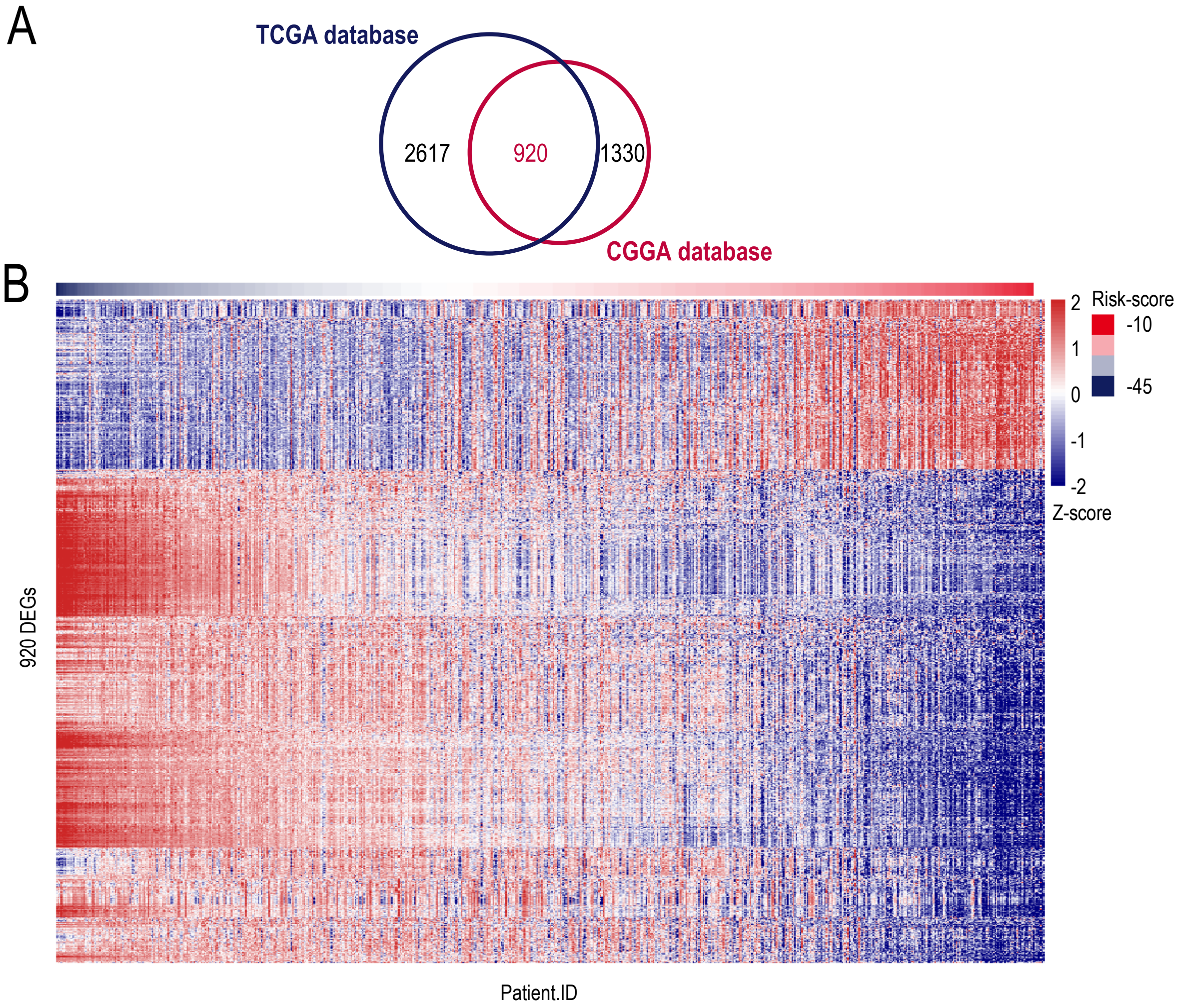

Fig. S7 Distinct gene expression associated with risk scores in patients with glioma. (A) Venn diagram showed the intersected differentialy expressed genes (iDEGs) from two independent datasets. (B) Heatmap showed 920 iDEGs in the TCGA database.

Fig. S8

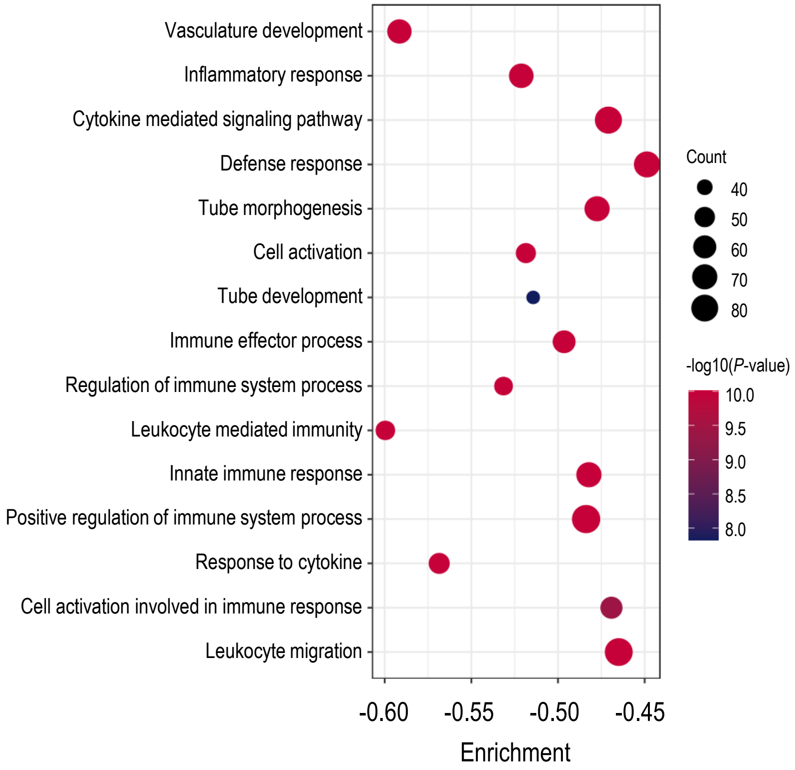

Fig. S8 GO analysis of 920 iDEGs high- and low-risk group showed enrichment of the immune response process.

Fig. S9

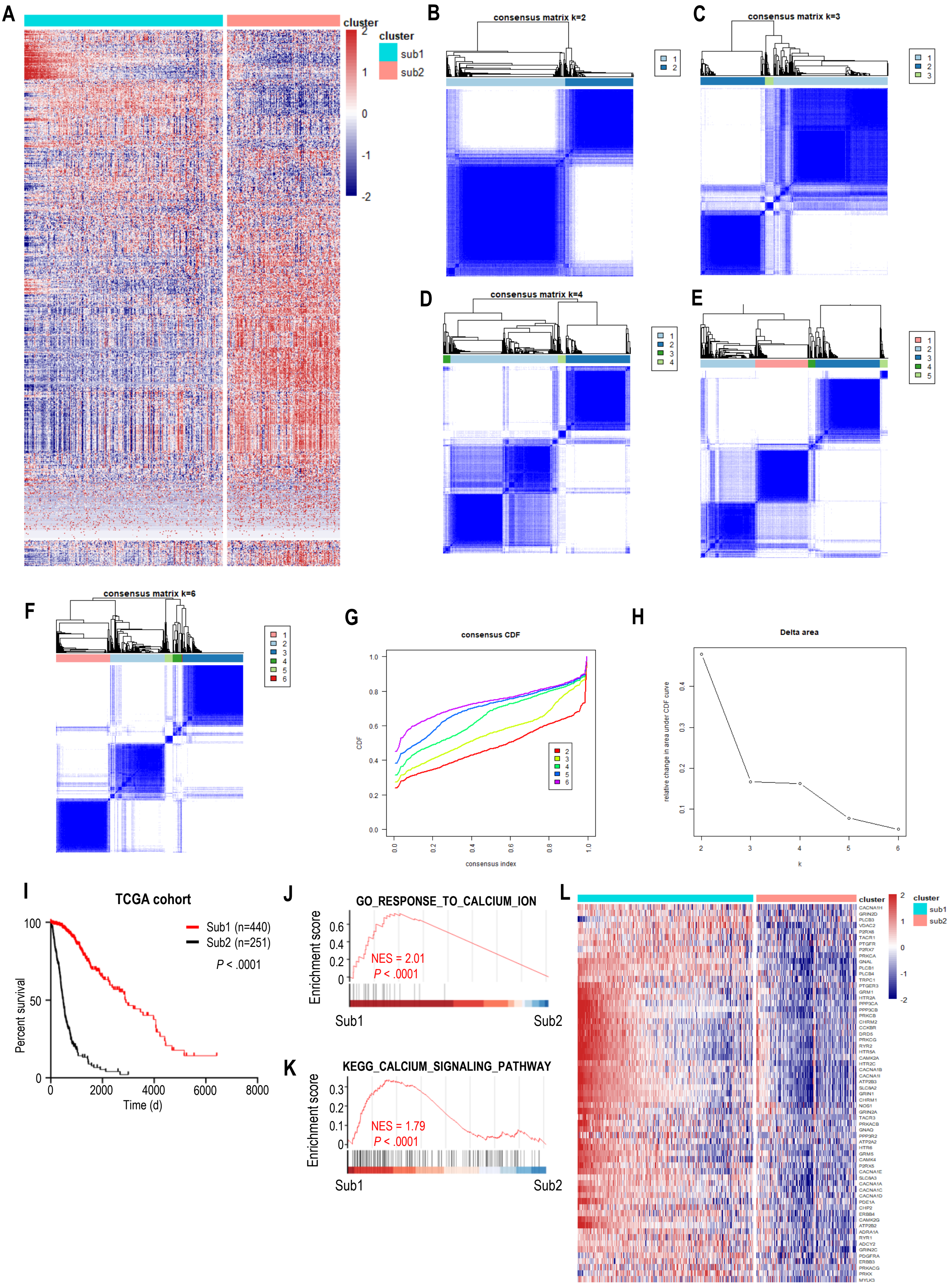
A
cluster
sub1
sub2
2
1
0
-1
-2
B
consensus matrix k=2
C
consensus matrix k=3
D
consensus matrix k=4
E
F
consensus matrix k=6
G
consensus CDF
CDF
consensus index
H
Delta area
relative change in area under CDF curve
k
I
TCGA cohort
Sub1 (n=440)
Sub2 (n=251)
P < .0001
Percent survival
Time (d)
J
GO_RESPONSE_TO_CALCIUM_ION
Enrichment score
NES = 2.01
P < .0001
Sub1
Sub2
K
KEGG_CALCIUM_SIGNALING_PATHWAY
Enrichment score
NES = 1.79
P < .0001
Sub1
Sub2
L
cluster
sub1
sub2

Fig. S9. The association between calcium signal and immune responses in TCGA glioma cohort.
(A) Heatmap of two subtypes defined by 2487 immune-related genes. (B-F) Consensus clustering based on the gene expression of 691 glioma in the TCGA cohort. Clustering matrix for k = 2 to k =6. (G) The cumulative distribution function (CDF) curves for k = 2 to k = 6. (H) Relative change in area under CDF curve for k = 2 to k = 6. (I) Kaplan-Meier analysis of two clusters based on overall survival (OS). (J-K) GO analysis (J) and KEGG analysis (K) indicated that the calcium signaling pathway enriched in the sub1 group of gliomas. (L) Heatmap showed that calcium-related genes significantly down-regulated in sub2 group of gliomas.
