# Supplementary material for: A Calcium-Related Immune Signature in Prognosis Prediction of Patients With Glioma
Source: Front Cell Dev Biol. 2021 Sep 28;9:723103. doi: 10.3389/fcell.2021.723103 (PMC8505737; doi:10.3389/fcell.2021.723103)
Supplement: Supplementary file 2 [file Data_Sheet_2.PDF]

Fig. R1, also showing as **Fig. S6** in revised manuscript

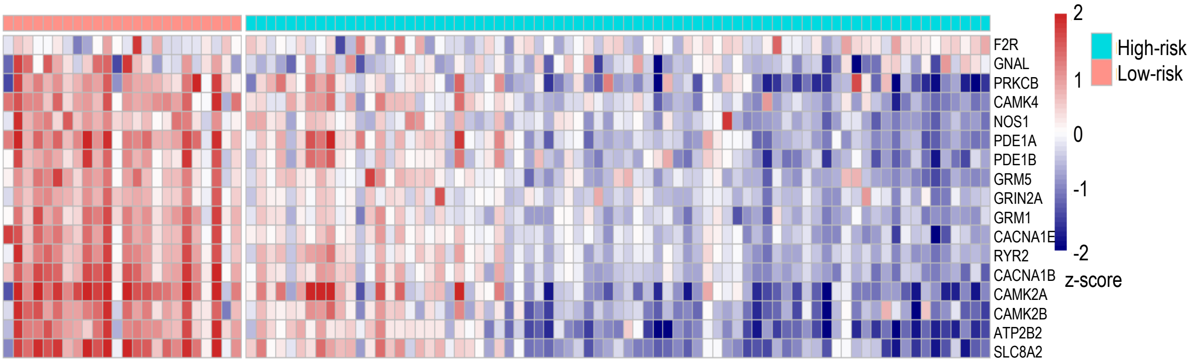

Fig. R1 (Fig. S6) Heatmap of the protein abundance by the proteome analysis showed differential expression between high- and low-risk groups in the dataset from Wang *et al*.

Fig. R2, also showing as **Fig. S9A** in revised manuscript

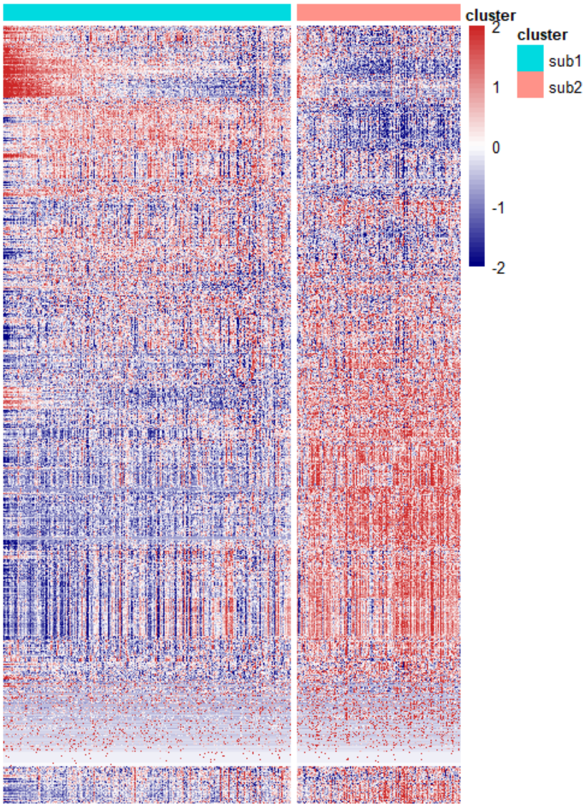

Fig. R2 (Fig. S9A) Heatmap of two subtypes defined by 2487 immune-related genes

Fig. R3, also showing as **Fig. S9B-S9H** in revised manuscript

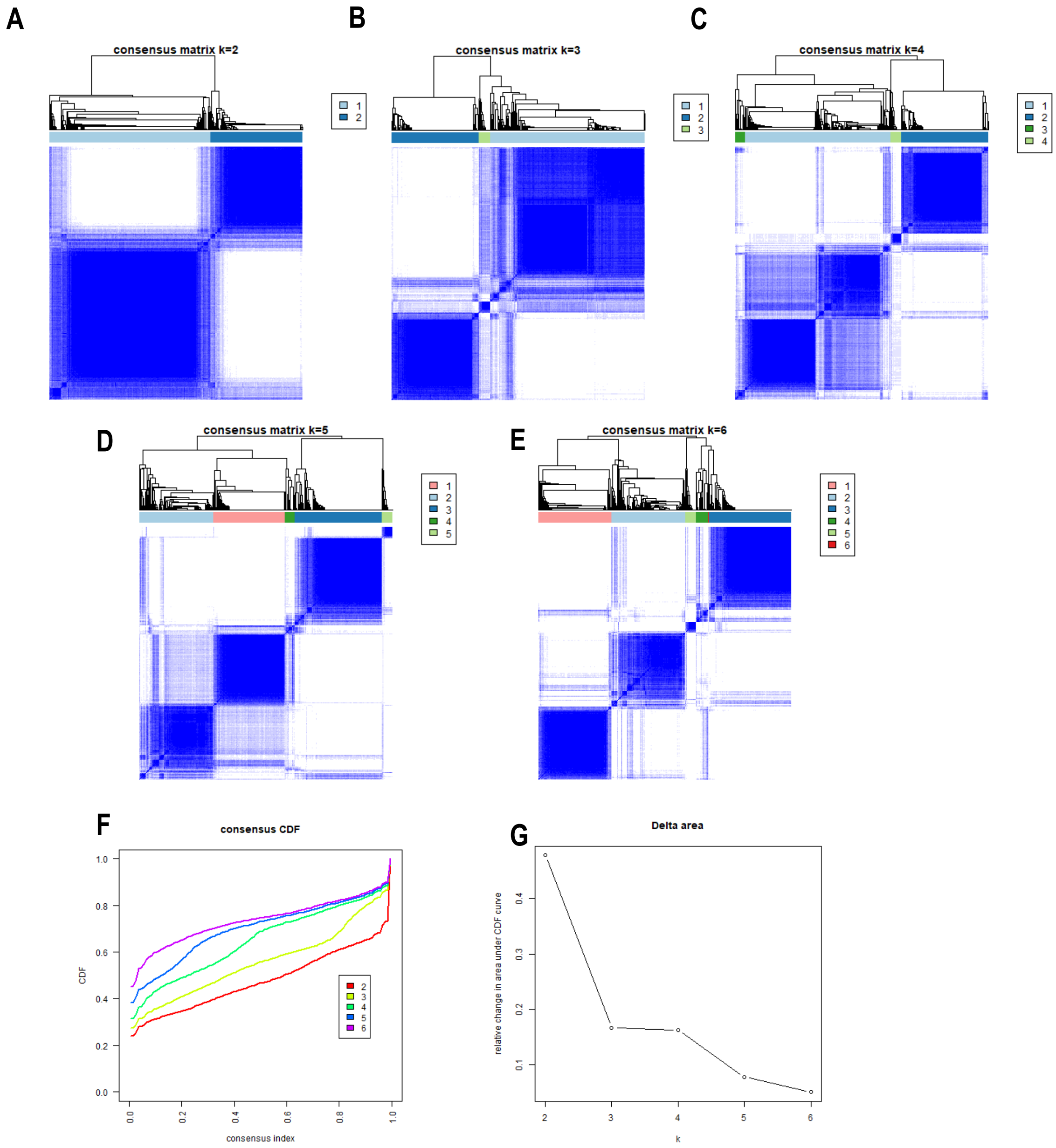

Fig. R3 (Fig. S9B-S9H) Consensus clustering based on the gene expression of 691 glioma in the TCGA cohort. (A–E) Clustering matrix for *k* = 2 to *k* =6. (F) The cumulative distribution function (CDF) curves for *k* = 2 to *k* = 6. (G) Relative change in area under CDF curve for *k* = 2 to *k* = 6.

Fig. R4 , also showing as **Fig. S9I** in revised manuscript

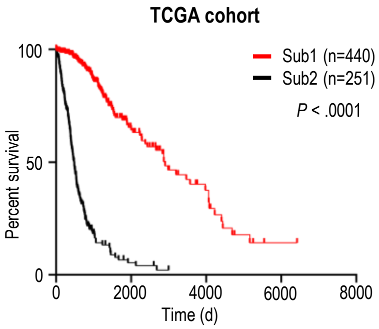

Fig. R4 (Fig. S9I) Kaplan-Meier analysis of two clusters based on overall survival (OS).

Fig. R5

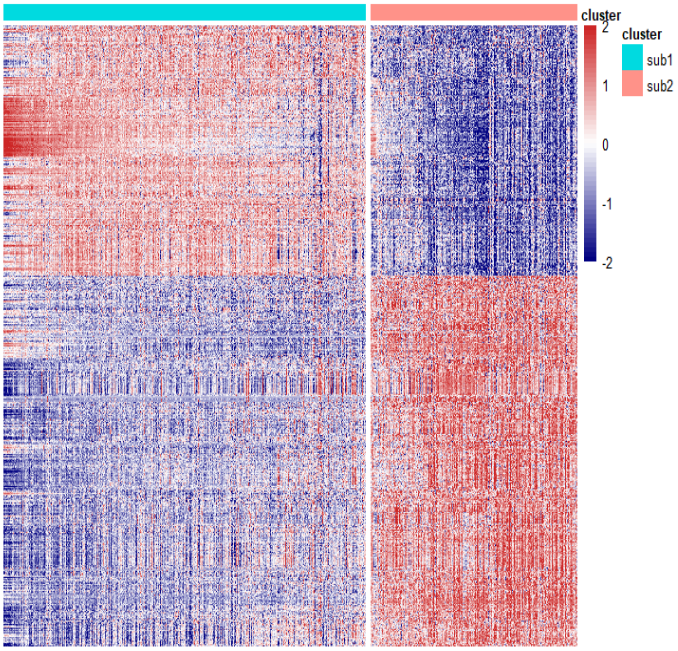

Fig R5. Heatmap showed the differential expressed genes (DEGs) between sub1 and sub2 ($|log_2FoldChange|>1$, p-value < 0.01).

Fig. R6, also showing as **Fig. S9J-S9L** in revised manuscript

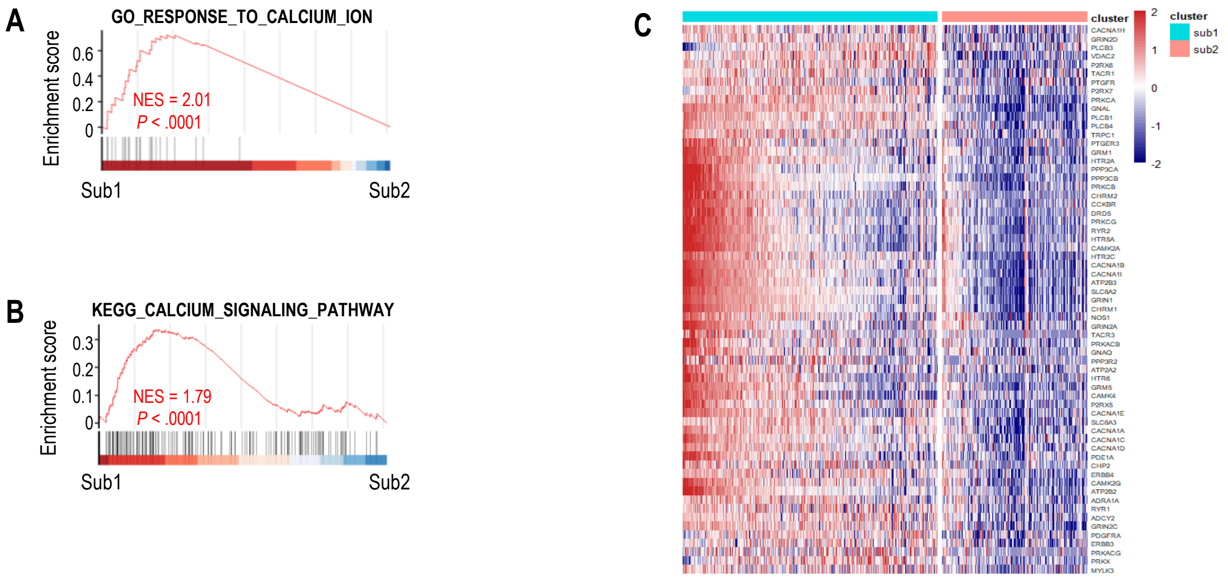

Fig R6 (Fig. S9J-S9L) Calcium signals varied greatly between two subtypes in glioma from TCGA database.

(A-B) GO analysis (A) and KEGG analysis (B) indicated that the calcium signaling pathway enriched in the sub1 groups of gliomas. (C) Heatmap showed that calcium-related genes significantly down-regulated in sub2 group of gliomas.
