# Supplementary material for: A Calcium-Related Immune Signature in Prognosis Prediction of Patients With Glioma
Source: Front Cell Dev Biol. 2021 Sep 28;9:723103. doi: 10.3389/fcell.2021.723103 (PMC8505737; doi:10.3389/fcell.2021.723103)
Supplement: Supplementary file 3 [file Data_Sheet_3.PDF]

Fig. RR1

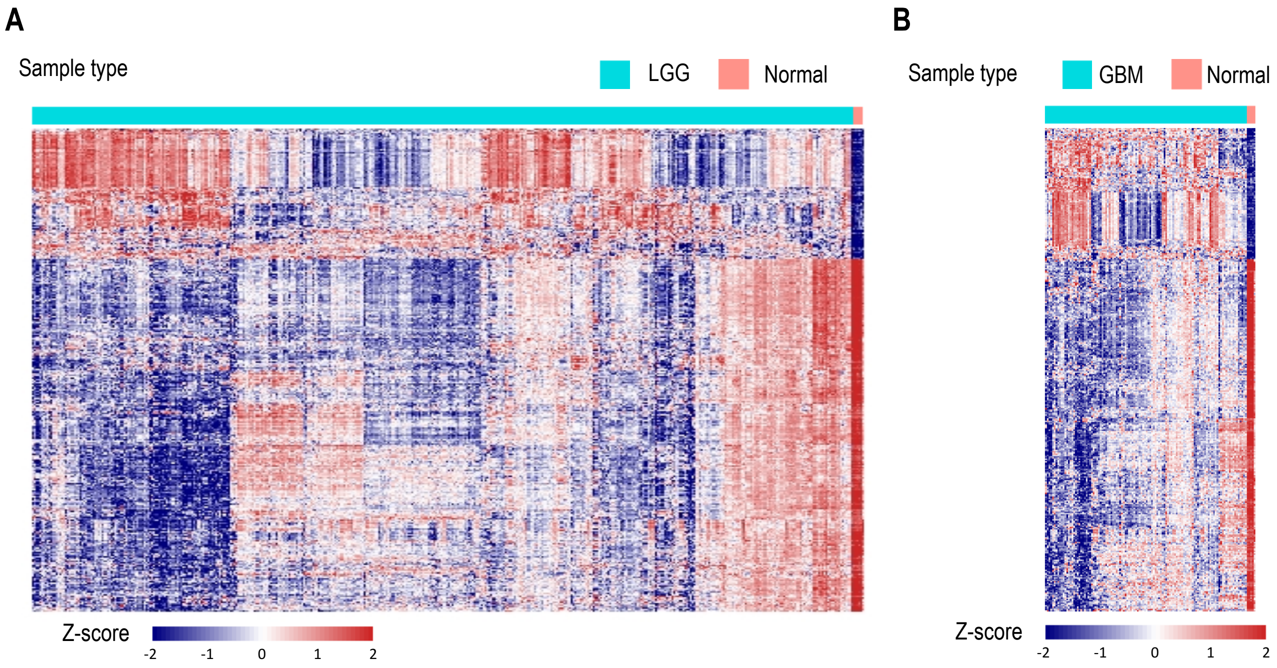

Fig. RR1
(A) Heatmap showed the 415 intersected differentialy expressed genes (iDEGs) in the lower grade glioma (LGG) cases (n = 525) and normal brain tissues (n = 5) from the TCGA database. (B) Heatmap showed the 415 iDEGs in the glioblastoma (GBM) cases (n = 166) and normal brain tissues (n = 5) from the TCGA database.

Fig. RR2

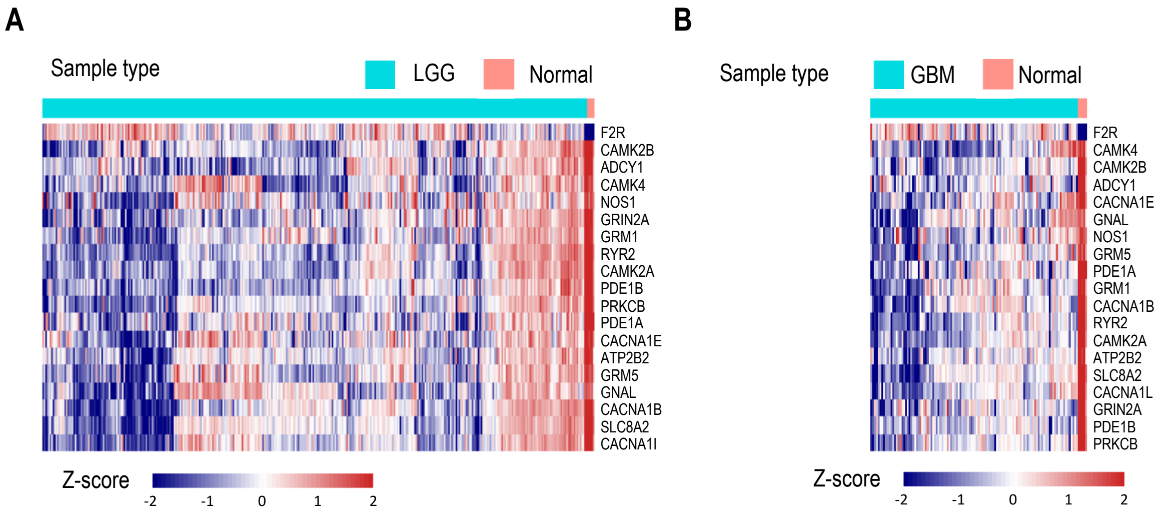

Fig. RR2

(A) Heatmap showed the 19 calcium-related genes in the LGG cases (n = 525) and normal brain tissues (n = 5) from the TCGA database.

(B) Heatmap showed the 19 calcium-related genes in the GBM cases (n = 166) and normal brain tissues (n = 5) from the TCGA database.
